# *Pectobacterium brasiliense*: Genomics, Host Range and Disease Management

**DOI:** 10.3390/microorganisms9010106

**Published:** 2021-01-05

**Authors:** Said Oulghazi, Sohaib Sarfraz, Maja A. Zaczek-Moczydłowska, Slimane Khayi, Abdelaziz Ed-Dra, Yassir Lekbach, Katrina Campbell, Lucy Novungayo Moleleki, Richard O’Hanlon, Denis Faure

**Affiliations:** 1 Department of Biology, Faculty of Sciences, Moulay Ismaïl University, BP.11201, Zitoune Meknes 50000, Morocco; s.oulghazi@yahoo.fr (S.O.); abdelaziz_iaa@yahoo.fr (A.E.-D.); 2 Institute for Integrative Biology of the Cell (I2BC), CEA CNRS University Paris-Saclay, 91190 Gif-sur-Yvette, France; 3 Department of Plant Pathology, Faculty of Agriculture, University of Agriculture Faisalabad, Faisalabad 38040, Pakistan; sohaib002@gmail.com; 4 Institute for Global Food Security, School of Biological Sciences, Queen’s University, Belfast BT9 5DL, UK; mzaczekmoczydlowska01@qub.ac.uk (M.A.Z.-M.); katrina.campbell@qub.ac.uk (K.C.); 5 Biotechnology Research Unit, CRRA-Rabat, National Institute for Agricultural Research (INRA), Rabat 10101, Morocco; slimane.khayi@inra.ma; 6 Shenyang National Laboratory for Materials Science, Northeastern University, Shenyang 110819, China; yassirlek@gmail.com; 7 Department of Biochemistry, Genetics and Microbiology, University of Pretoria, Pretoria 0002, South Africa; lucy.moleleki@fabi.up.ac.za; 8 Agri-Food and Biosciences Institute, 18a Newforge Lane, Belfast BT9 5PX, UK; r.ohanlon@qub.ac.uk; 9 Department of Agriculture, Food and the Marine, D02 WK12 Dublin 2, Ireland

**Keywords:** *Pectobacterium brasiliense*, soft rot, blackleg, *Solanaceae*

## Abstract

*Pectobacterium brasiliense* (Pbr) is considered as one of the most virulent species among the Pectobacteriaceae. This species has a broad host range within horticulture crops and is well distributed elsewhere. It has been found to be pathogenic not only in the field causing blackleg and soft rot of potato, but it is also transmitted via storage causing soft rot of other vegetables. Genomic analysis and other cost-effective molecular detection methods such as a quantitative polymerase chain reaction (qPCR) are essential to investigate the ecology and pathogenesis of the Pbr. The lack of fast, field deployable point-of-care testing (POCT) methods, specific control strategies and current limited genomic knowledge make management of this species difficult. Thus far, no comprehensive review exists about Pbr, however there is an intense need to research the biology, detection, pathogenicity and management of Pbr, not only because of its fast distribution across Europe and other countries but also due to its increased survival to various climatic conditions. This review outlines the information available in peer-reviewed literature regarding host range, detection methods, genomics, geographical distribution, nomenclature and taxonomical evolution along with some of the possible management and control strategies. In summary, the conclusions and a further directions highlight the management of this species.

## 1. Introduction

The group of bacterial plant pathogens known as pectinolytic, soft rot Pectobacteriaceae (SRP) consists of two genera: *Pectobacterium* and *Dickeya* [1]. These pathogens are responsible for plant tissue maceration resulting in water-soaked lesions which lead to collapse of the infected tissue, wilting and death of the plants [2,3]. *Pectobacterium brasiliense* (Pbr) is a worldwide-distributed bacterial plant pathogen causing soft rot of a wide range of economically important crops. Pbr has been reported in Eurasia: from Western European countries to Russia and China [4,5,6], in Africa: South Africa, Morocco, Algeria and Kenya [7,8,9,10]. Pbr has been constantly reported since 2004 causing significant losses particularly of potato (*Solanum tuberosum* L.) [11,12,13,14] and posing a threat to worldwide potato gross production value which is estimated to be $63914 million in 2018 [15]. Significant disease caused by Pbr has been pointed out in Belgium, the Netherlands and Switzerland: for instance, in 2015 in Switzerland prevalence of Pbr reached 70% of the samples collected from potato field outbreaks [13]. In Russia, in 2017 more than 20% soft rot disease was caused by Pbr and this species also has been reported as a predominant cause of blackleg of potato in the Moscow region in 2018 [14]. However, in some other countries such as the United Kingdom and Norway, so far Pbr is not considered as a problematic species in potato fields [16,17,18]. In addition to losses in potato production, Pbr causing loses of other crops: for instance, in the years 2014–2015, in five Chinese provinces (Shandong, Shanxi, Hebei, Henan, and Liaoning), Pbr caused soft rot of cucumber whereby the disease incidence was vary from 15 to 50% in different fields, causing 20 to 30% yield losses [6]. Hence, Pbr is considered to be a highly aggressive pathogen and is causing more soft rot/blackleg disease outbreaks elsewhere than other *Pectobacterium* species [4,19].

This review summarizes current information on Pbr in genomics (especially its contribution to advances in taxonomy, diversity, pathogenic traits), host range and disease management (including Pbr detection methods and control strategies).

## 2. Genomics: From Advances in Taxonomy to Insights in Biodiversity and Virulence

During the last two decades, high-throughput sequencing (HTS) technologies have been an important driving force in the progress of life sciences [20]. Genomic information has also been one of the cores of molecular biology in studying evolutionary biology, taxonomy, phylogeny, and it can also provide insights to distinct niches adaptation [21,22]. The availability of several published genomes illuminates new strategies for exploring the genomic datasets, through comparative genomics approaches. Therefore, some genomic comparisons have also allowed for delineating taxa, for identifying the core repertoire of virulence genes shared by related organisms and, for locating gene clusters or genome islands exclusive of species or even unique of a strain for developing molecular tools for identification and detection of pathogens [23]. To date, few comparative studies have also been conducted regarding *Pectobacterium* species [24,25,26,27,28]. Until now, six Pbr complete genome sequences are publicly available: Pbr PCC21 (misnamed as *P. carotovorum* PCC21 in NCBI) isolated from *B. rapa* ssp. *pekinensis* [29], Pbr BZA12 from *Cucumis sativus* [25], Pbr BC1 from *Brassica rapa* ssp. *pekinensis* [30], Pbr SX309 from *C. sativus* fruit [31], Pbr HNP201719 and Pbr 1692 from *S. tuberosum* [32]. In addition, more than thirty draft genomes of Pbr species have also been deposited in the GenBank genome database.

**Figure 1:**
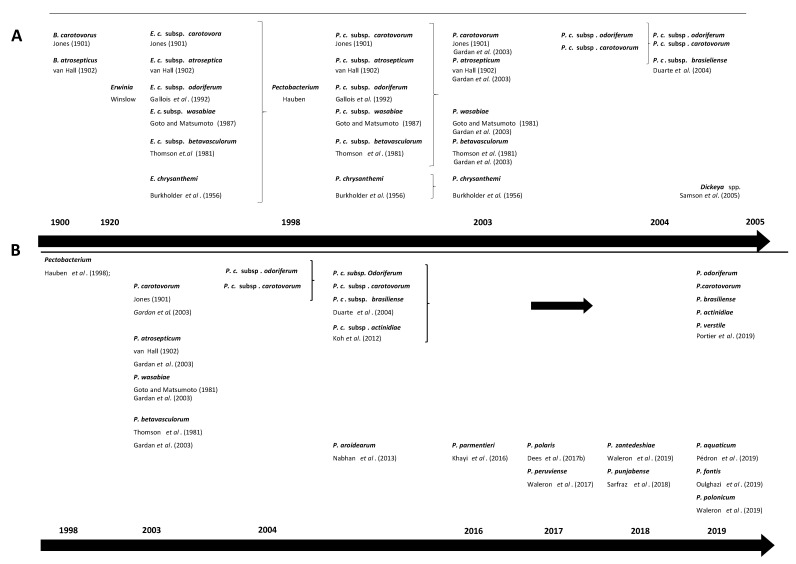
Timeline changes in taxonomy of *Pectobacterium* spp. (**A**) changes from early described species causing soft rot symptoms *Bacillus carotovorus* and *Bacillus atrosepticus*, through establishment of *Erwinia* species by Winslow et al. up to re-classification of *Pectobacterium* and *Dickeya* spp. by Samson et al. [11,33,40,46,47,51,52,53,54,55] (**B**) changes from re-classification of *Pectobacterium* spp. up to most recent proposed species [11,33,34,38,46,47,51,52,55].

Over the past decades, the taxonomy of the genus *Pectobacterium* has undergone major modifications (Figure 1) [33,34,35,36,37,38,39,40,41,42,43,44,45,46,47,48]. Establishment of Pbr as a separate taxon was initially based on amplification of the intergenic spacer region in PCR, differences in 16S rRNA gene sequence, and analysis of biochemical traits [11]. Later, it was proposed as a subspecies of *P. carotovorum* through multi-locus sequence analysis (MLSA) in several studies [49,50]. This taxon has been recently elevated at a species level Pbr using a combination of phylogenomics, *in-silico* DNA-DNA hybridization (isDDH) (70% threshold), average nucleotide identity (ANI) (95–96% threshold) and biochemical traits [34]. In the same paper, the authors also described *P. odoriferum*, *P. actinidiae* and *P. versatile* as new species, and emended description of *P. carotovorum*. Because of this recent change, we have used the updated species description *P. carotovorum*, but we alert the readers that some of these *P. carotovorum* strains may potentially belong to *P. versatile*.

**Figure 2:**
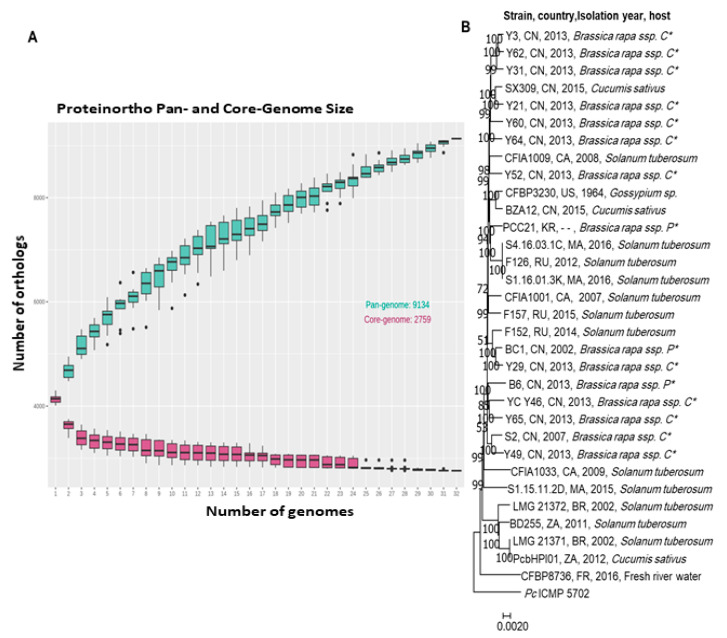
The genomic comparison of the available Pbr genomes in NCBI: (**A**) core and pan genome analysis, (**B**) phylogenomic analysis of the concatenated core genes of 30 Pbr strains was conducted using Proteinortho V.6 [56]. The genes were clustered based on the criteria of 50% identity on at least 50% of the length of the alignment, the *P. carotovorum* ICMP5702 was used as out of the group. (P *: Pekinensis; C *: Chinensis).

Pbr species are diverse and less homogeneous than other *Pectobacterium* spp. Indeed, the ANI Pbr species threshold is 95% while the ANI species threshold is often 96% for other *Pectobacterium* spp. Aside from improvement in Pbr species delineation, comparative genomics gives an insight into gene richness in the Pbr genomes. The analysis of the Pan-genome and Core-genome of 30 Pbr strains was conducted using the software Proteinortho V6 [56]. The genes were clustered based on the criteria of 50% identity on at least 50% of the length of the alignment. The analysis highlighted an accumulative number of 8210 gene families within the 30 strains analyzed (Figure 2A).

The pan-genome curve indicates an exponential increase in size over adding new strains stating an open pan-genome. Based on this result, we could predict an expansion of the unique gene pool by the addition of new Pbr genomes. In contrast, the pan-genome, gene number in the core-genome decreased upon addition of new strains to reach a total of 2968 core genes. Remarkably, the phylogenetic tree generated from concatenated core-genome genes showed no clear congruence between the evolutionary relationships of the Pbr isolates and the geographical origin or plant host of their isolation (Figure 2B). The relationships between the genome diversity of Pbr isolates and aggressiveness on plant hosts should be experimentally investigated using plant assays.

Comparative genomics revealed highly conserved virulence genes in the *Pectobacterium* [28]. Some of these virulence factors include, amongst other quorum-sensing (QS), secretion systems, adhesion, plant cell wall-degrading enzymes (PCWDEs), motility, chemotaxis, siderophores and biofilm formation [30,31,57,58,59]. Similarly, knowledge of the phylogeny and the genetic basis for the pathogenicity of *Pectobacterium* was expanded recently, as 84 *Pectobacterium* genomes were screened for the presence of 159 genes that are known as virulence factors [30]. Only a few studies have focused on studying the complete genome of Pbr, and consequently, the pathogenicity and the mechanisms for genetic adaptation to the host remains largely unknown [31]. The genomic analysis of Pbr (reported as *P. carotovorum* subsp. *brasiliense* SX309) showed the presence of many similar virulence factors already described in *Pectobacterium* spp. including the PCWDE biosynthetic genes, bacterial QS genes, secretion system genes, chemotactic genes and flagella [28,31]. In Pbr SX309 several variable regions of two subtype CRISPR-Cas immune systems and type VI secretion system may also contribute in the infection process [31]. A study conducted by Moleleki et al. [58] showed that Pbr colonization, swimming motility, and flagella biosynthesis are also regulated by the QS system. In addition, Pbr 1692 is known to be more aggressive and typically outcompetes other members of SRP [60]. The production of several antimicrobial compounds by Pbr 1692 could contribute to its capacity to effectively colonize different types of ecological niches [61].

## 3. Symptoms, Host Range and Geographical Distribution

**Figure 3:**
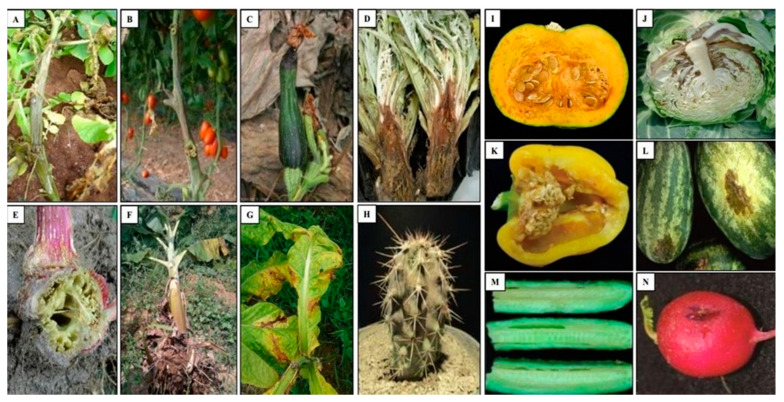
Symptoms caused by Pbr in several plant species. (**A**) Potato (*S. tuberosum*), (**B**) Tomato (*Solanum lycopersicum*), (**C**) Zucchini (*Cucurbita pepo*), (**D**) Artichoke (*Cynara cardunculus* var. *scolymus*), (**E**) Amaranth (Amaranthus), (**F**) Banana (*Musa* sp.), (**G**) Tobacco (*Nicotiana tabacum*), (**H**) Tetecho (*Neobuxbaumia tetetzo*), (**I**) Squash (*Cucurbita pepo*), (**J**) Cabbage (*Brassica oleracea*), (**K**) Pepper (*Capsicum annuum*), (**L**) Watermelon (*Citrullus lanatus*), (**M**) Cucumber (*Cucumis sativus*), (**N**) Raphanus (*Raphanus sativus*) [6,7,63,64,82,85,86,87].

Bacteria belonging to the genus *Pectobacterium* were considered among the most threatening of phytopathogens to the health of vegetable, ornamental and fruit crops, including Pbr [11,62]. Pbr is responsible for the degradation of the cell wall of several plant hosts and causing blackleg and soft rot diseases (Figure 3).

Indeed, Pbr infects a wide range of plant species, including both monocotyledon and dicotyledon clades [7,11,62,63,64,65]. Nowadays, 19 different plant species belonging to 10 families are reported as hosts of Pbr (Table 1). As Pbr was isolated in different continents, it has apparently adapted to many environments and climates, including tropical and temperate regions. Therefore, this pathogen could be more widespread than currently known.

The family *Solanaceae* is considered as the major host of Pbr. Five host plant species belonging to this family have been reported so far, including potato (*S. tuberosum* L.), tomato (*S. lycopersicum*), pepper (*C. annuum*), eggplant (*S. melongena*), and tobacco (*N. tabacum*) (Table 1). On the basis of available studies reporting the presence of Pbr in the host plants, more than 50% of them have been associated with potato (this study, Table 1). In fact, Pbr has been reported in potato in Brazil since 2004 [11], followed by its detection in North America [66,67,68], Europe [12,13,14,69,70], Africa [7,9,71,72], Asia [62,73,74,75,76], and New Zealand [77]. Generally, Pbr causes blackleg and soft rot in potato leaves, stems, and tubers [8,10,66,68,74]. Tomatoes have been reported to be also infected by Pbr, resulting in a large loss for producers. In Colombia, Jaramillo et al. [78] reported the presence of Pbr in ‘Calima’ hybrid tomato, causing aqueous and brown lesions on the lower stem, necrosis of the vascular bundles, and some plants presented cracking symptoms along the stem. In Florida, Pbr infected “heirloom” tomatoes, causing wilting, necrosis of leaves and stems, and water-soaked pith tissue [79]. Pbr caused brown water-soaked, soft-rotted pith tissue, and internal vascular discoloration in Grafted tomato plants from Sicily (Italy) [80]. In the pepper, Pbr caused water-soaked, necrotic tissues, and wilt with defoliation in Venezuela [81], fruit decay and pedicel decay in Korea [82], and black spot disease in China [83]. Pbr causes soft and aqueous legions in eggplant from South Korea [62], as well as necrosis, drying and rotting in the leaves of Tobacco from China [84].

The plant families *Cucurbitaceae* and *Brassicaceae* are also affected by Pbr. Infection of cucumber was reported in China [6] and South Africa [90], and was characterized by the appearance of gummosis on the surface of leaves, stems, petioles, and fruits, dark brown coloration of the basal stems, as well as yellow spots could sometimes emerge at the edge of infected leaves [6]. The infection of zucchini were reported in Poland [69], Serbia [65], Austria [91], Brazil [92], and Italy [88], and was characterized by water-soaked lesions and fruits macerating. Infection of squash and watermelon were reported in Northern Serbia [65] with disease appearing in squash as light brown, slightly sunken, soft, and macerated tissue with the presence of a water-soaked lesions. Infection caused by Pbr in watermelon developed as a soft rot brownish lesions on the infected stems. Furthermore, soft rot in cabbage and Chinese cabbage were reported in Poland [69], and South Korea [62], characterized by water-soaked lesions on leaves and gray to pale brown discoloration of tissues. However, root rot disease of *Raphanus* was reported in China [93], in which the infected plants were characterized by yellowish foliage, blackened center leaves, and decayed roots.

*Asteraceae* and other host families like *Amaranthaceae*, *Chenopodiaceae*, *Cactaceae*, *Nepenthaceae*, *Malvaceae*, *Primulaceae*, and *Caricaceae* were rarely infected by Pbr. In Sicily (Italy), the infected Artichoke (*Cynara cardunculus* var. *scolymus*) develops wilting of the older leaves accompanied by dark-green to dark-brown soft rotting of the pith [63]. In a study carried out in the USA, infected sugar beet (*B. vulgaris*) was characterized by soft decay of internal root tissues, blackening of petiole vascular bundles, half-leaf yellowing, and frothing [94]. Moreover, Amaranth (*Amaranthus* L.) grown in South Korea has been infected by Pbr presenting typical soft rot symptoms like wilting, defoliation and odd smell [86]. In Mexico, the infection of Tetecho (*N. tetetzo*) by Pbr causes damages of the whole plant, as well as collapse and disintegration [85]. Pbr is also known to cause asymptomatic infections in some plant species.

Recently, a survey was conducted in potato fields where the infected plants were detected, showing the isolation of Pbr from asymptomatically infected *Malva nicaeensis* [95]. However, the mechanism of infection in asymptomatic hosts is not well understood.

Soft rot diseases caused by Pbr are rare in monocotyledon hosts. Only one study has reported the presence of Pbr in hosts belonging to monocotyledon clade. This study isolated the pathogens from different cultivars of banana (*Musa* sp.) in India and the French overseas territory Martinique [64,88], in which the pathogen causing rhizome rot was characterized by disagreeable foul-smelling and internal decay of the pseudostem [64].

Although Pbr can infect plants from both monocotyledons and dicotyledons, it can present some specialization toward the host at the strain level. In fact, nine strains of Pbr isolated from cucumber were in vitro tested against different plants species. These strains caused soft rot in potato, tomato, green pepper, broccoli, radish, mustard, zucchini, cucumber and others, but did not cause disease in balsam pear and loofah [6]. In another study, strains of Pbr isolated from *N. tetetzo* caused soft rot on many plant species, including *Myrtillocactus geometrizans*, *Opuntia ficus-indica*, *S. lycopersicum*, *Cucumis sativus*, and *Daucus carota* subsp. *sativus*, but they did cause symptoms on *C. pepo*, *Physalis ixocarpa*, and *Brassica oleracea* var. *capitata* [85].

Moreover, Pbr has been isolated from non-host environment, including water in Spain and the rhizosphere of *S. dulcamara* in France [34,88]. Pbr is considered a major threat for horticulture crops in these regions, as can be easily transmitted to other fields through irrigation water systems or by its persistence in the soil.

## 4. Isolation, Characterization and Detection of Pbr

Visual assessment of blackleg and soft rot disease symptoms of infected plants is not enough to confirm the presence of Pbr, as the symptoms are indistinguishable from infection caused by other SRP. Hence, the use of accurate detection and identification tools is required to study the biodiversity and pathogenicity of Pbr.

### 4.1. Pbr Isolation

Symptomatic plants should be collected and stored in a cool container until they are received in the lab, and their diseased parts processed to isolate SRP. Naas et al. [9] collected tuber samples from the margins of diseased tissue of potato, and after sample maceration in sterile water, streaked material on nutrient agar plates, incubated at 27 ± 1 °C. Gillis et al. [81] used fruits, stems and leaves from infected pepper (*C. annuum* L.) to isolate pathogens while other microbes were eliminated by surface sterilizing tissues for 30 s in 75% ethanol. Other researchers have used 0.6% sodium hypochlorite (NaOCl) for 30 s [96], 0.6% NaOCl for 1 min [97], and 70% ethanol for 30 s followed by 0.5% NaOCl for 30 s [83]. The streaked agar plates are incubated at optimal temperatures (26–28 °C) for Pbr growth for different incubation times (48 to 78 h) depending on the medium.

Several non-selective media are used for the culture of Pbr, such as nutrient agar, tryptic soy agar and Luria-Bertani agar [62]. Besides, other semi-selective-diagnostic media can be used in order to detect, isolate and enumerate SRP. One of the most used media for this purpose is crystal violet pectate (CVP) medium and its modifications [98,99]. This is a semi-selective medium that contains pectin as a carbon source and crystal violet as an inhibitor for the growth of Gram-positive bacteria. SRP are secreting PCWDEs, which metabolize pectin, resulting in formation of characteristic cavities in this solid medium [100]. From its first use by Cuppels and Kelman [101], this medium was modified and improved in order to provide a better pectin source that can be easily degraded by bacteria. For example, Hélias et al. [100] tried six different pectin sources. Among them, they found that AG366 pectin was highly effective in CVP medium compared to commercial ones. The characteristic of this medium makes it suitable for identification of SRP. It provides better results as it allows formation of deep cavities due to degradation of pectin by microbes [100].

### 4.2. Characterization by Biochemical Methods

The most of biochemical tests are used as additional methods for confirmation of identity and genetic grouping of *Pectobacterium* including Pbr [9]. Biochemical assays used for identification and differentiation of Pbr including Gram staining, the ability of isolates to produce oxidase and catalase [85], carbon source utilization [34,102], acid production from maltose and α-methyl-D-glucoside, growth at 29 °C and 37 °C, tolerance to 5% NaCl, erythromycin sensitivity, indole production, lactose fermentation, erythromycin sensitivity and gas production from D-glucose [6]. The Hugh and Leifson medium was employed to study the oxidative or fermentative metabolism of glucose [68]. Furthermore, King’s B medium was used to evaluate the ability of Pbr isolates to produce fluorescent pigments [81]. One of the commercial tests available for this purpose is GN2 microplate which is designed for the characterization and identification of different aerobic Gram-negative bacteria including Pbr [6,62].

### 4.3. Characterization and Detection by Molecular Methods

Several molecular method have been employed for the characterization of Pbr, including conventional polymerase chain reaction (PCR) [8,9], real-time qPCR [103], PCR-restriction fragment length polymorphism (PCR-RFLP) [10], pulsed-field gel electrophoresis (PFGE), MLSA [62] and HTS [8], with conventional PCR and real-time PCR commonly used in laboratories for robust confirmation of Pbr in the samples [9,103] (Table 2).

The most recommended method for differentiation of Pbr from other SRP are MLSA and HTS enable to confirm phylogenetic position within *Pectobacterium* genus [8,34]. Additionally, among other reported detection methods (Table 2), MLSA is widely used due the availability of the data in the public data base (NCBI). Also, it was used for taxonomic information [34,111] and the study of the diversity of Pbr with the related species [8,9]. Most recently, several housekeeping genes were reported to differentiate Pbr in MLSA schemes [8,9,34,50]. For example, Nabhan et al. [50] have used eight housekeeping genes in MSLA to delineate species of *Pectobacterium*, including glutamylphosphate reductase (*proA*), aconiate hydrase 1 (*acnA*), mannitol-1-phosphate 5-dehydrogenase (*mtlD*), isocitrate dehydrogenase (*icdA*), malate dehydrogenase (*mdh*), glucose-6-phosphate isomerase (*pgi*), glyceraldehyde-3-phosphate dehydrogenase A *(gapA*), and the RNA polymerase subunit sigma factor 38 (*rpoS*). However, as these methods highly efficient for differentiation of Pbr are time consuming and need qualified personnel and specialized equipment to perform analysis.

### 4.4. Other Methods

In contrast to other widely distributed SRP (i.e., *P. atrosepticum*, *P. carotovorum* and *Dickeya*) any of sensitive and robust POCT detection methods such as isothermal amplification or handled biosensor device have not been developed thus far as specific for detection of Pbr. These reported POCT methods are specific for *P. atrosepticum* [112], *P. carotovorum* [113], *P. aroidearum* [114] or *Dickeya* spp. [115,116,117]. However, fairly recently interesting approach has been reported by Ahmed et al. [118] for detection of *Pectobacterium* spp. including Pbr. Developed assay based on recombinase polymerase amplification adopted on lateral-flow device (RPA-LFD) was reported as sensitive (10 fg/μL) and specific handled device detecting *Pectobacterium* spp. including Pbr. Additionally, the benefits coming from the use of this POCT method is performing a test directly from infected plant material with no need for DNA extraction [118]. Recently, the use of infrared spectroscopy and machine learning has been proposed as a cheaper (relative to molecular techniques) method of identifying and differentiating between different genera, species and strains of SRP [119].

## 5. Management and the Control of Pbr

Methods for the control of Pbr have not been explored in as much detail as the other SRP species (i.e., *P. carotovorum* or *Dickeya* spp.) [19]. Recent management strategies for SRP have focused on physical pre-treatments of seeds, good hygiene and phytosanitary regulation, however new discoveries in genetics, biocontrol and nanoscience show promise for controlling pathogens such as Pbr [120,121,122]. Thus far, limited in vitro approaches have been tested to control Pbr (Table 3) [123,124,125,126].

There has been, however, an extensive amount of research on biocontrol of the most widespread economically important SRP, especially *Dickeya solani*, *P. carotovorum* and *P. atrosepticum* [5,134,135,136,137,138,139,140]. In fact, biocontrol of SRP using bacteriophages has progressed the most among other methods, with a stage where formulated commercial product is available, including Biolyse^®^, which was demonstrated to be efficacious at preventing soft rot in packaged potatoes caused by *Pectobacterium* and *Dickeya* species [141].

### 5.1. Preventive Measures

Pbr is currently a regulated species in Egypt, and is regarded as not present in the jurisdiction, with phytosanitary actions taken on findings [127]. There are no other national or international phytosanitary regulations specifically for the control of Pbr, however a range of other regulatory measures and hygiene practices are used to limit the spread of this and other species of SRP. SRP pathogens in Europe are regulated under EU and national legislation on plant health and seed marketing. The EU Plant Health Regulation [142] and its associated legislation [143] contains measures to limit the spread of *Dickeya* and *Pectobacterium* species that are capable of causing blackleg in potato as Regulated Non-Quarantine Pests (RNQP). An RNQP is a pest that is mainly transmitted through specific plants for planting, its presence on those plants for planting has an unacceptable economic impact as regards the intended use of those plants. In order to limit the spread of these RNQPs, the National Plant Protection Organization must put in place steps including inspection, testing and where needed phytosanitary measures to destroy non-conforming plants and seed crops. There is zero tolerance for *Dickeya* or *Pectobacterium* species in EU pre-basic potato seed, increasing to 1% and 4% for basic and certified seed, respectively. In order to be marketed as certified planting material in the EU under the potato seed marketing directive [144], seed potatoes are tested for blackleg (i.e., caused by a number of SRP) the NPPO in the country of production [127]. Seed potatoes cannot have more than 2% by weight of soil attached to the tubers [144]. Seeds of other vegetable hosts of SRP are also regulated [128], with a view to prevent the spread of plant pathogens in seed, but this directive is rather vague in the specified measures to be applied, stating that “Diseases and harmful organisms which reduce the usefulness of the seed shall be at the lowest possible level.

It has been known for many years that physical contact between infected and uninfected plants and tubers, or damp storage conditions for harvested tubers are responsible for spreading potato soft rot [145]. Good hygiene practices when handling, planting and storing potato tubers is still strongly recommended for controlling potato soft rot and blackleg [129]. Though not specifically designed to control Pbr, these phytosanitary measures and hygiene measures such as use of certified seed, washing and disinfection of material and equipment, are known to decrease the losses of vegetables due to SRP [146].

### 5.2. Chemical Control

Over the decades, the outlook was taken to use synthetic chemicals including antibiotics, bactericides, organic, inorganic salts, fertilizers (i.e., calcium and nitrogen) and peptides to control SRP, however none of these studies reported the control particularly of Pbr [147,148]. Several reports indicated promising results, with reduction of soft rot and blackleg incidences in potato. For example, efficient control of SRP was demonstrated using antibiotic combinations such as streptomycin/oxytetracycline hypochlorite, streptomycin/mercury compounds, kasugamycin and virginiamycin [147] or bactericides (acetic acid, boric acid and bleaching powder) [148]. Small molecular weight defense proteins named as antimicrobial peptides (AMPs) are another group of interesting compounds with proven antibacterial properties against a wide range of pathogens including bacteria, fungi and viruses [149], but have also been reported to inhibit the growth of SRP and reduce soft rot symptoms in vitro [150,151]. With the extent of synthetic chemical compounds (incl. antibiotics) to be used in agriculture, there is a risk that these products might be hazardous to humans and/or the environment, or could lead to the development of antimicrobial resistance [152,153,154]. Additionally, these methods are not considered to be fully efficient to control SRP in the potato sector [150,155].

### 5.3. Nanoparticles

It is commercially and scientifically attractive to develop a control method targeting a broad range of SRP, and most recently, close attention has been given to products with high versatility which could be adapted targeting several bacterial species causing soft rot [156]. Nanoparticles are also perspective components which could be used as an antibacterial agent such as silver nanostructures (AgNPs) which have been shown to be antiseptic against 650 species of pathogenic bacteria, fungi and viruses and could be combined with other antagonistic bacteria. This component has already been tested against SRP species including Pbr and shown high antiseptic properties against both *Dickeya* and *Pectobacterium* spp. [121,130,131,132].

### 5.4. Biocontrol Using Bacteriophages

Only relatively few bacteriophages have been showed to infect Pbr species. Two broad host range lytic bacteriophages: vB_PcaP_PP2 (PP2) and T-4 Myoviridae PM2 phage were isolated from soil samples infected two *Pectobacterium* species: *P. carotovorum* and Pbr under host range screening [125,126]. Moreover, genomic analysis of PM2 and PP2 phages, indicated genes important in biocontrol such as lysis genes (i.e., detected endolysins lyses bacterial cell wall), which can be useful as an antimicrobial agent or engineered and applied as suitable for potential biocontrol application in the future [125,126]. Two other lytic bacteriophages: *Podoviridae* PP99 and *Myoviridae* PP101, were demonstrated to be specific only against Pbr with the potential to be used as a biocontrol agent [19]. In vitro experiments performed by Czajkowski et al. [134,135] reported successful suppression of disease symptoms caused by *D. solani*, *P. carotovorum* and *P. wasabiae* on potato tubers. Soleimani-Delfan et al. [157] reported the isolation of two bacteriophages from the Caspian Sea active against *D. dadanti* (the causative agent of potato soft rot in Iran), which reduced rot when inoculated onto *Geranium* spp. by approximately 89% in comparison to the positive control. Marei et al. [158] pointed out the reduction of soft rot symptoms on potato tubers inoculated with pathogen *P. carotovorum* and bacteriophage Pc1 isolated in Egypt. Buttimer et al. [159] reported significant reduction of soft rot symptoms through whole tuber assays by isolated bacteriophages CB1, CB3, and CB4 active against *P. atrosepticum*, the causative agent of blackleg in western Europe. Carstens et al. [136] reported significant suppression of soft rot caused by *P. atrosepticum* using a phage cocktail containing six selected bacteriophages on potato tubers in simulated storage conditions. The phage efficacy to control Pbr in field trials has not been reported so far [19], however, recently the efficacy of single bacteriophages or phage cocktails to control *P. carotovorum* and *P. atrosepticum* was proven under field conditions [14,138,139].

### 5.5. Biocontrol Using Bacteria

The species *Pseudomonas fluorescens* and *Pseudomonas putida* have been shown to be efficacious against SRP. Raoul des Essarts et al. [160] combined in vitro assays with tuber maceration assays and potato plant protection assays in greenhouse, to show that *P. fluorescens* and *P. putida* strains and their mix inhibited the growth and symptom incidence of *Dickeya* spp. and *Pectobacterium* spp. and could therefore be used as a biocontrol of SRP. Genome analysis of these *Pseudomonas* strains revealed a potential production of the antibacterial cytostatic metabolite paerucumarin, as well as siderophore operons. The species *Bacillus subtilis* has also been shown to be an effective biocontrol: Sharga and Lyon [161] showed that *B. subtilis* provided in vitro protection of potato tubers against *P. atrosepticum* and *P. carotovorum*. Following this, several studies have indicated the protective effect of *B. subtilis* and *Bacillus amyloliquefaciens* against SRP, on Chinese cabbage, lettuce and carrots [62,162,163]. Other *Bacillus* taxa, including *Bacillus* E-65 and *B. licheniformis* have also proven to be efficacious against SRP [164,165]. The bacterium *Paenibacillus dendritiformis* was also shown to have potential as a biocontrol agent for SRP of potato [166] through in vitro, greenhouse and field trial studies. *Lactobacillus* spp. are also active against SRP through the production of organic acids such as propionic or lactic acid [167,168]. Other lactic acid-producing bacteria also show potential to be used a biocontrol agent for SRP, including *Leuconostoc mesenteroides*, *Leuconostoc citreum*, *Weisella cibaria*, *Lactococcus lactis* and *Enterococcus mudti* [167]. The approach reported by Maciag et al. [156] relay on the use of a cocktail containing antagonistic bacteria species (*Serratia plymuthica* strain A294, *Enterobacter amnigenus* strain A167, *Rahnella aquatilis* strain H145, *Serratia rubidaea* strain H440, and *S. rubidaea* strain H469), targeting a mix of *Dickeya* and *Pectobacterium* spp. This broad range biocontrol, significantly reduced soft rot incidences and severity in simulated storage conditions over six months of the experiment [156].

The use of *Bdellovibrio* and like organisms (BALO) as ‘living antibiotics’ in the medical field has grown dramatically [164]. However, the potential to use of these organisms in the control of phytopathogens is beginning to emerge. A relevant example for control of Pbr is that which was demonstrated by Youdkes et al. [133], showing that BALO strains are highly effective against Pbr on potato slice assays.

### 5.6. Biocontrol Targeting Quorum-Sensing (QS): Chemicals and Biostimulants

Prospective approaches known for the control of SRP, rely on interfering with the virulence of Pbr and other *Pectobacterium* spp. regulated by the QS system via quorum-quenching (QQ) approaches [169,170,171,172,173]. QQ impairs production PCWDEs [123]. In the study reported by Joshi et al. [123] two essential oils produced by plants: carvacrol and eugenol interfere with QS gene expression, biofilm formation and production of PCWDEs leading to suppression of the infection caused by Pbr in vitro. Another report provided by Joshi et al. [123] showed the effectiveness to use two phenolic acids: SA and CA to interfere with the QS system. An interesting approach to interfering with the QS of other SRP, was to use gamma-caprolactone (GCL), a biostimulant of the growth of QQ-bacteria, which are able to disrupt QS-signal of *Pectobacterium* spp. [174]. Application of GCL to potatoes grown using aeroponics led to an increase in the relative abundance of the QQ-population, including bacteria of the *Rhodococcus erythropolis* species, indicating that one possible way to control soft rot would be to apply a treatment in the nutrient supply of vegetables grown aeroponically [175]. In *R. erythropolis*, GCL also increases transcription of the gene encoding a lactonase that disrupts *Pectobacterium* QS-signal [176]. Most of these experiments focus on in vitro approach in laboratory settings, and there is still a lack of extended field trials to demonstrate the applicability of these methods.

## 6. Further Directions

Similar to other SRP, virulence of Pbr is regulated by QS which stimulates secreting PCWDEs, toxins-like proteins (promoting plant cell-death), molecules, large proteins (serine protease, hemolysin and hemagglutinin) and unknown effectors through six secreting systems, i.e., except Pbr, T1S was confirmed to be present in *P. atrosepticum* and *P. carotovorum* which are not considered as emerged species within SRP. Therefore, further transcriptomic studies of Pbr enable to more deeply understand its pathogenicity, virulence and aggressiveness.

Most recently recommended approaches for detection SRP including Pbr are time consuming and expensive identification methods such as MLST analysis with concatenated housekeeping genes (e.g., *gyrB*, *recA*, *secY*), while only whole genome sequencing would give an answer on the presence of specific genes responsible for virulence or resistance in these particular isolates. As only three sets of PCR primers (conventional and real-time qPCR) reported thus far are specific for detection of Pbr, fast, reliable and cost-effective POCT method (i.e., biosensor, LFA or isothermal amplification assay), which could be useful for differentiation Pbr from other *Pectobacterium* spp. would be beneficial to develop. Moreover, undeniable potential for detection of aggressive species such as Pbr and i.e., *Dickeya* spp. in one assay would have miniaturized multiplex device such as microarray. Such handheld devices would be useful for detection of Pbr. for Institutes, Agriculture Departments or farmers in the face of increase number of disease outbreaks caused by Pbr. Thus, future research should also include development of new POCT methods to manage Pbr and other SRP. Moreover, there is still need to discover novel, cost-effective, environmentally and human safe, control strategies for Pbr and other SRP pathogens. The deficiency of effective and safe chemical control strategies make biocontrol a promising tool for reducing losses of vegetables, especially through their use in integrated pest management programs. Novel biocontrol strategies are underlined through high-budget projects funded by agriculture departments, research funding bodies and commercial companies carried out worldwide and in Europe to minimize losses caused by SRP pathogens (i.e., using bacteriophages) (AHDB 2019; APS Biocontrol 2019; PCA, 2019; NCBiR 2018). Although bacteriophage based microbial pest control agents (MPCA) are not yet common in agriculture, this biocontrol method as the first reach the stage of commercialization (APS Biocontrol 2020). However, adaptation of new biocontrol technologies which are effective under laboratory conditions for i.e., amassing sufficient phage/bacteria quantities need further progress to scale from lab to industrial use (i.e., several biocontrol productions failed due to underestimating costs associated with developing and marketing microbial products). Further work on commercialization to more microbial products could became available to control SRP should also involve collecting data on its efficacy, stability, and safety to satisfy EU regulations on plant protection products (1107/2009/EC). As not enough data is available, further work should also include more research about ecotoxicity of microbial products on the surrounding ecosystem, animals and food security.

## 7. Conclusions

This review has presented the most recent information about Pbr. The scientific evidence and plant health information indicates that Pbr is a serious threat to plant health globally, as the pathogen threatens the health of several important vegetable and fruit crops. There is currently no efficacious control method for Pbr and a lack of understanding of its ecology and epidemiology, therefore the full threat caused by Pbr is not clear, as yet. Consequently, in order to limit damage provoked by Pbr, there is a need for a deeper exploration of its genomics, biology and etiology to improve soft rot disease detection caused by Pbr and new effective management strategies to be commonly implemented in agriculture practice.

## Figures and Tables

**Table 1:** Hosts range, disease symptoms and geographical distribution of Pbr.

Clade	Family	Host (Plant Species)	Region/Country	General Disease	Symptoms	References
**Dicotyledons**	*Solanaceae*	Potato (*Solanum tuberosum*)	Brazil, Kenya, Japon, Canada, South Africa, Switzerland, Poland, New Zealand, South Korea, Netherlands, Algeria, Turkey, Russia, China, Egypt, USA, Hawaii, Thailand, Morocco, Zimbabwe, Syria, France	Soft rot and blackleg	The infected plants were stunted with yellowish foliage, water-soaked regions with watery ooze, darkened and necrotic basal stem symptoms extending upward.	[7,8,9,11,12,13,14,62,66,67,68,69,70,71,72,73,74,75,76,77,88,89]
		Tomato (*Solanum lycopersicu* )	South Korea, Colombia, USA, Italy	Stem rot	Soft and aqueous lesions, dark brown discoloration of the basal part of stem petioles, water-soaked pith tissue and internal necrotic.	[62,78,79,80]
		Pepper (*Capsicum annuum*)	Venezuela, South Korea, China	Soft rot and black spot	Watery lesions at the basal part of the stem, water-soaked and necrotic tissue, defoliation and fruit decay.	[81,82,83]
		Eggplant (*Solanum melongena*)	South Korea	Soft rot	Water-soaked lesions, soft rot symptoms on fruits, discoloration of vascular tissues, stem hollowness, and dark green lesions that turned brown with age.	[62]
		Tobacco (*Nicotiana tabacum*)	China	Bacterial leaf blight	Necrosis along the main or lateral veins, drying and rotting of the leaves.	[84]
	*Cucurbitaceae*	Cucumber (*Cucumis sativus*)	China, South Africa	Soft rot	The gummosis emerged on the surface of leaves, stems, petioles, and fruit. The basal stem color was dark brown and the stem base turned to wet rot. Yellow spots and wet rot emerged at the edge of the infected leaves and gradually infected the leaf centers.	[6,90]
		Zucchini (*Cucurbita pepo*)	Poland, Brazil, Serbia, Austria, Italy	Soft rot	Round water-soaked lesions. The affected tissues were light brown, slightly sunken, soft, and macerated.	[65,69,88,91,92]
		Watermelon (*Citrullus lanatus*)	Serbia	Soft rot	Soft rot brownish lesions of stems, collapse and wilting of entire vines.	[65]
	*Brassicaceae*	Cabbage (*Brassica oleracea* var. *capitata*)	Poland	Soft rot	Symptoms were characterized by gray to pale brown discoloration and expanding water-soaked lesions on leaves.	[69]
		Chinese cabbage (*Brassica rapa* ssp. *pekinensis* and *chinensis*)	South Korea	Soft rot	Water-soaked lesions, affected tissue turns brown and becomes soft and mushy. Leaves, stems, and roots may decay entirely.	[62]
		Raphanus (*Raphanus sativus*)	China	Root rot	The infected plants were stunted with yellowish foliage and blackened center leaves and the infected roots exhibited a completely decayed pith region.	[93]
	*Asteraceae*	Chrysanths (*Chrysanthemum*)	South Korea, France, USA	Soft rot	*	[62,88]
		Artichoke (*Cynara cardunculus* var. *scolymus*)	Italy	Soft rot	Chlorosis and wilting of the older leaves accompanied by dark-green to dark-brown soft rotting of the pith.	[63]
	*Amaranthaceae*	Sugar beet (*Beta vulgaris*)	Poland, USA	Soft rot	Soft decay of internal root tissues, reddening of affected tissue after cutting, blackening of petiole vascular bundles, half-leaf yellowing, and frothing.	[69,94]
	*Chenopodiaceae*	Amaranth (*Amaranthus*)	South Korea	Soft rot	Wilting, defoliation and odd smell.	[86]
	*Cactaceae*	Tetecho (*Neobuxbaumia tetetzo*)	Mexico	Soft rot	Soft rot that damages the whole plant and causes its fall and disintegration.	[85]
	*Nepenthaceae*	Nepenthes (*Nepenthes*)	South Korea	Soft rot	*	[62]
	*Malvaceae*	Bull Mallow (*Malva nicaeensis*)	Israel	**	**	[95]
		*Gossypium* sp.	USA	*	*	[88]
	*Primulaceae*	*Cyclamen* sp.	France	*	*	[88]
	*Caricaceae*	*Carica papaya*	France (Overseas territory, La Réunion)	*	*	[88]
**Monocotyledon**	*Musaceae*	Banana (*Musa* sp.)	India, France (Overseas territory, Martinique)	Rhizome rot	Massive soft rot accompanied by disagreeable foul-smelling rot of the rhizome and internal decay of the pseudostem as the infection spread upward.	[64,88]
**Non-Host Environment**	Water		Spain	*	*	[34,88]
	Rhizosphere of *Solanum dulcamara*		France	*	*	[88]

* Symptoms were not described, ** Symptomless.

**Table 2:** Isolation and detection methods of Pbr.

Classification	Method	Applications	Primers/Probes	Features	Target Species	References
Artificial media	CVP, modified CVP (single or double layer), enrichment using PEB, other formulations	Isolation pure cultures of bacteria	Pectinase	Degradation of polypectate or other reaction	*Pectobacterium* and *Dickeya* spp.	[100,101,104,105,106,107,108]
PCR methods	Conventional	Identification to genus level	Y1 & Y2	*pel* gene	*Pectobacterium* spp. including Pbr	[109]
		Identification to species level	BR1f/L1r	16S–23S rRNA	Pbr	[11]
	Real-time (qPCR)	Identification and quantification	Pb1F/Pb2R; Probe name PbPr	(16S-23S ITS) and tRNA-Glu gene AraC sequences	Pbr	[4,103]
			PbrFW/PbrRv; Probe name Pbrb			
DNA Sequencing methods	Single gene sequencing	Identification of species	gapA326F/gapA845R	*gapA* gene	*Pectobacterium* and *Dickeya* spp.	[110]
			mdh86F/mdh628R	*mdh* gene	*Pectobacterium* spp.	[9]
			Pec.dnaA-F1/Pec.dnaA-R1	*dnaA* gene	*Pectobacterium* spp.	[66]
	Whole genome sequencing (MLSA, ANI, isDDH, phylogenomics)	Identification of species	Not applicable	Not applicable	The study of phylogenetic relationships of species within a genus	[8]

**Table 3:** Summary of control methods reported to manage Pbr.

Control/Management Method	Agent	Target Species	Application	Information	References
**Preventive Measures**	Not applicable	SRP including Pbr	In the course of potato production and storage	Seed potato tubers are tested for the presence of SRP causing blackleg under seed certification scheme. Good hygiene practice when planting, harvesting and storing potato tubers prevent the spread of SRP and soft rot disease development.	[127,128,129]
**Nanoparticles**	AgNPs	SRP including Pbr	In vitro	AgNPs tested in laboratory conditions showed high antiseptic properties against SRP.	[121,130,131,132]
**Biocontrol**	*Bdellovibrio* spp.	Pbr	Potato slice bioassay	In bioassay experiment on potato slices *Bdellovibrio* spp. were tested with high efficacy against Pbr.	[133]
	Salicylic acid (SA) and cinnamic acid (CA)	Pbr	Bioassays on potato and *Calla lily*	In laboratory and bioassays experiments SA and CA interfered with QS and significantly suppressed Pbr growth.	[123]
	Carvacrol and eugenol	Pbr	Potato, cabbage and *C. lily* bioassays	Two tested compounds have interfered with QS gene expression and caused on biofilm formation and secreting PCWDEs leading to effective suppression of infection caused by Pbr.	[124]
	Bacteriophages PP99 and PP101	Pbr	Host range screening	Two isolated phages PP99 and PP101 were tested as highly specific against Pbr which potentially could be used for biocontrol application.	[19]
